# Gene mutation landscape of a rare patient with acute megakaryoblastic leukemia after treatment of intracranial germ cell tumor

**DOI:** 10.3389/fonc.2023.1093434

**Published:** 2023-05-09

**Authors:** Li-Xin Wang, Wei-Jie Liao, Yu-Hua Jiang, Chao Chen, Wang-Sheng Lu, Feng Yin, Hao-Yong Ning

**Affiliations:** ^1^ Department of Hematology and Oncology, International Cancer Center, Shenzhen Key Laboratory of Precision Medicine for Hematological Malignancies, Shenzhen University General Hospital, Shenzhen University Clinical Medical Academy, Shenzhen University Health Science Center, Shenzhen, China; ^2^ Department of Hematology, Navy General Hospital, Beijing, China; ^3^ Department of Neurosurgery, Tiantan Hospital, Beijing, China; ^4^ Department of Thoracic Surgery, Peking University Shenzhen Hospital, Shenzhen Peking University-The Hong Kong University of Science and Technology Medical Center, Shenzhen, China; ^5^ Department of Neurosurgery, Navy General Hospital, Beijing, China; ^6^ Department of Pathology, Navy General Hospital, Beijing, China

**Keywords:** acute megakaryoblastic leukemia, intracranial germ cell tumor, gene mutation, tumor origin, whole exome sequencing

## Abstract

**Introduction:**

It was first reported that germ cell tumor patients suffer from hematologic malignancies 37 years ago. Since then, the number of relevant reports has increased each year, with most cases being mediastinal germ cell tumor. Theories have been proposed to explain this phenomenon, including a shared origin of progenitor cells, the effects of treatment, and independent development. However, up to now, no widely accepted explanation exists. The case with acute megakaryoblastic leukemia and intracranial germ cell tumor has never been reported before and the association is far less known.

**Methods:**

We used whole exome sequencing and gene mutation analysis to study the relationship between intracranial germ cell tumor and acute megakaryoblastic leukemia of our patient.

**Results:**

We report a patient who developed acute megakaryoblastic leukemia after treatment for an intracranial germ cell tumor. Through whole exome sequencing and gene mutation analysis, we identified that both tumors shared the same mutation genes and mutation sites, suggesting they originated from the same progenitor cells and differentiated in the later stage.

**Discussion:**

Our findings provide the first evidence supporting the theory that acute megakaryoblastic leukemia and intracranial germ cell tumor has the same progenitor cells.

## Introduction

Cancer is now one of the leading causes of human mortality, with its incidence and mortality rates growing each year. There were 474,519 new cases of leukemia, 308,102 new cases of brain and nervous system cancers, and 74,458 new cases of testicular cancer, which accounted for 4.5% of all cancers, in 2020. Despite this relatively low percentage, these types of cancer still resulted in a large number of deaths (approximately 572,257) in 2020. Hence, in order to guide the development of new therapies for cancers that occur in the blood, testis, brain, and nervous system, it is crucial to further study the detailed pathogenesis (1).

Germ cell tumor (GCT) is a malignant tumor that predominantly affects young people and typically originates from the gonad (2, 3). However, 2%-5% of cases occur outside the gonad, primarily in the mediastinum, and very few appear in the central nervous system (GCT-CNS) (4–6). GCT-CNS mostly occurs in the midline of the CNS in individuals between 1 and 30 years old. GCT patients are reported to suffer from hematologic malignancies (HM), including AML, ALL, and MDS, and the link between GCT and HMs was first discovered in 1985 (7–9). Generally, GCT appears before HMs, and most of the associated cases are mediastinal GCT (10). There are some hypotheses about the relationship between HMs and mediastinal GCT. For instance, pluripotent primordial germ cells and hematopoietic stem cells have common precursor cells in the early embryo, and HM may be related or unrelated to the treatment of mediastinal GCT (11–15). However, the specific mechanisms are still elusive. For subsequent concurrent acute megakaryoblastic leukemia M7 (AML-M7) of intracranial GCT, which have not been previously investigated, the specific pathogenesis is not well understood. Therefore, revealing the pathological relationship between intracranial GCT and AML-M7 for effective treatment is of great importance.

Herein, we report a patient with an intracranial germ cell tumor and acute megakaryoblastic leukemia, which has never been reported previously. Using whole-exome sequencing (WES), we identified that both tumors harbored the same driver genes and mutation sites, indicating that both tumors shared the same origin and differentiated in a later stage. Our findings provided the first evidence supporting the theory that acute megakaryoblastic leukemia and intracranial GTC arise from the same progenitor cells.

## Materials and methods

### Ethics statement

The pathological and genetic images and results used in this article have been approved by the Navy General Hospital Medical Ethics Committee and informed consent has been given by the patient’s family member.

### Formalin-fixed paraffin-embeddingof intracranial GCT sample, bone marrow sample preparation, and genomic DNA extraction

Intracranial GCT tissues were obtained from surgical resection and immediately fixed with formalin. The tissues were then embedded with paraffin and stored. Prior to DNA extraction, the FFPE sample was dewaxed, digested with protease K, and then vortexed to decrosslinking.

Bone marrow samples were collected from the AML-M7 patient and DNA was isolated using the MagMAX™ DNA extraction kit (A36570, Thermo Fisher Scientific) using Nucleic acid extractor KingFisher Duo Prime (Thermo Fisher Scientific). The DNA was then assessed for quality using a Nanodrop 2000.

### DNA library construction and WES

Genomic DNA samples were sheared using an ultrasonic crusher (Covaris) to be randomly interrupted into 180-280bp fragments. The ends of the fragments were repaired and A-tails were added. Both ends of the fragments were then connected to adaptors to prepare the DNA library using the SureSelect Human All Exon kit (Agilent). After pooling with a specific index, the DNA library was hybridized with biotin-labeled probes in a liquid phase. The exons were finally captured using streptomycin magnetic beads. The library was then sequenced using Illumina Hiseq sequencing platform.

### Workflow of mutation analysis pipeline for WES

The sequencing data processing and variant detection pipeline is shown in **Figure S1**. Reads containing sequencing adapters and low-quality reads were removed using fastp software (16). Then, the high-quality data of each sample was mapped to the human HG19 reference genome with Megabolt software. To ensure accurate variant calling, local realignment around Indels and base quality score recalibration were performed using GATK4 (17). The sequencing depth and coverage for each sample were then calculated based on the alignments. SNVs and Indels were detected using the GATK4 software. These mutations were annotated with ANNOVAR (18) and filtered for common mutations in the database to remove potential germline variants and obtain somatic mutations. A mutation was considered somatic if the frequency of the variant was less than 0.5% at dbSNP, 1000 Genomes (1000G) database (http://www.1000genomes.org), Exome Sequencing Project (ESP) 6500 database (http://evs.gs.washington.edu/EVS), Exome Aggregation Consortium (ExAC) database (http://exac.broadinstitute.org), or GnomAD database.

## Results

### Intracranial germ cell tumors of the case were diagnosed

**Figure 1:**
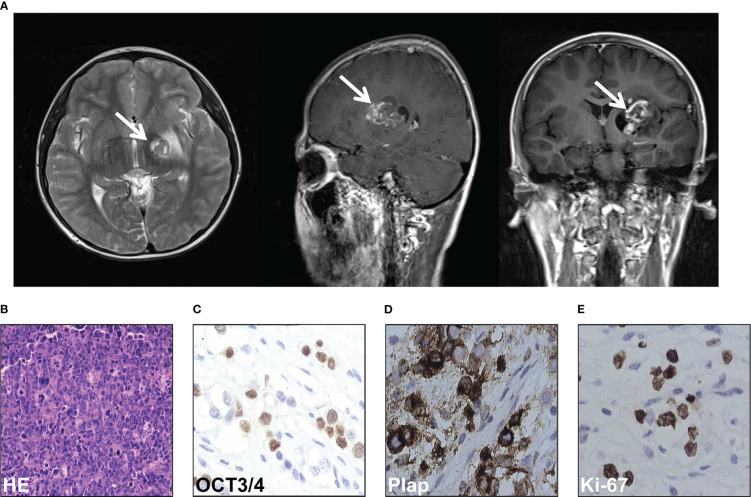
Intracranial germ cell tumors of the case were diagnosed. **(A)** Computed tomography scan of the brain of the case. **(B)** HE staining of the pathological tissues of surgical resection. **(C-E)** Immunohistochemical stain of OCT3/4 **(C)**, Plap **(D)**, and Ki-67 **(E)** of the pathological tissues of surgical resection.

An 11-year-old boy presented with increasing weakness in his right leg in September 2012. On neurological examination, right hemiparesis was discovered and he obtained a Glasgow coma score of 15/15. Computed tomography (CT) of the brain showed a 4cm×5.5cm mass in the left basal ganglia region (**Figure 1A**). A whole-body CT scan did not reveal any masses in other parts of the body. His serum α-fetoprotein and β-human chorionic gonadotropin (HCG) were both markedly elevated (87.99 ng/ml and 6.58 mIU/mL respectively). Given the elevated markers and CT scan features, a diagnosis of germ cell tumor or glioma was considered in October 2012. Due to the technical difficulty, the neurosurgical team recommended that the tumor should be shrunk with chemotherapy first. Therefore, he received two cycles of teniposide (35mg/m^2^ days 1 to 3), cisplatin (25 mg/m^2^ days 1 to 3), and ifosfamide (2g days 1 to 3) for two months, from October to December 2012. He responded well to the treatment and tolerated it. His level of β-HCG normalized, and the AFP markedly decreased to 10 ng/ml. Surgical resection was then carried out three months after GCT diagnosis, in January 2013. Histopathologic examination showed a mixed germ cell tumor including immature teratoma, yolk sac tumor, and germ cell tumor (**Figure 1B**). Immunohistochemical staining of the tissue was positive for OCT3/4, Plap, and Ki-67 (**Figures 1C−E**).

### Acute megakaryocytic leukemia of the case was diagnosed

**Figure 2:**
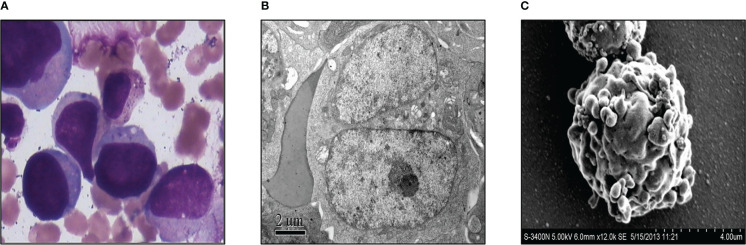
Acute megakaryocytic leukemia of the case was diagnosed. **(A)** Morphological examination of bone marrow with microscope. **(B)** Transmission electron microscopic examination of the bone marrow. **(C)** Bone marrow was exanimated by scanning electron microscope.

Three weeks after surgery, the patient received a third cycle of similar chemotherapy, followed by local radiotherapy in March 2013 upon completion of chemotherapy. Three months after surgery for GCT, in April 2013, he presented with fever, and a full blood count revealed severe thrombocytopenia and mild anemia, but a normal white blood cell (wbc) count (wbc 4.2×10^9^/L, Hb 102g/L, platelet count 16×10^9^/L). Peripheral blood film examination revealed blast cell, leading to a referral to our hospital for further investigation. A bone marrow examination was performed, and the morphology of the bone marrow was consistent with acute megakaryocytic leukemia, with 36% of blasts out of the total nucleated cells (**Figure 2A**). Transmission electron microscopic examination showed large blasts with irregular shapes, a big nucleus, and increased mitochondria (**Figure 2B**). Furthermore, under the scanning electron microscopic examination, the blasts had multiple projections from the membrane (**Figure 2C**). Flow cytometry analysis showed that the abnormal blast was positive for CD117, CD71, CD38, CD9, and CD36^dim^ and negative for other markers. These results were consistent with acute megakaryocytic leukemia or AML-M7.

### Multiple chromosomal abnormalities of bone marrow and intracranial GCT

**Figure 3:**
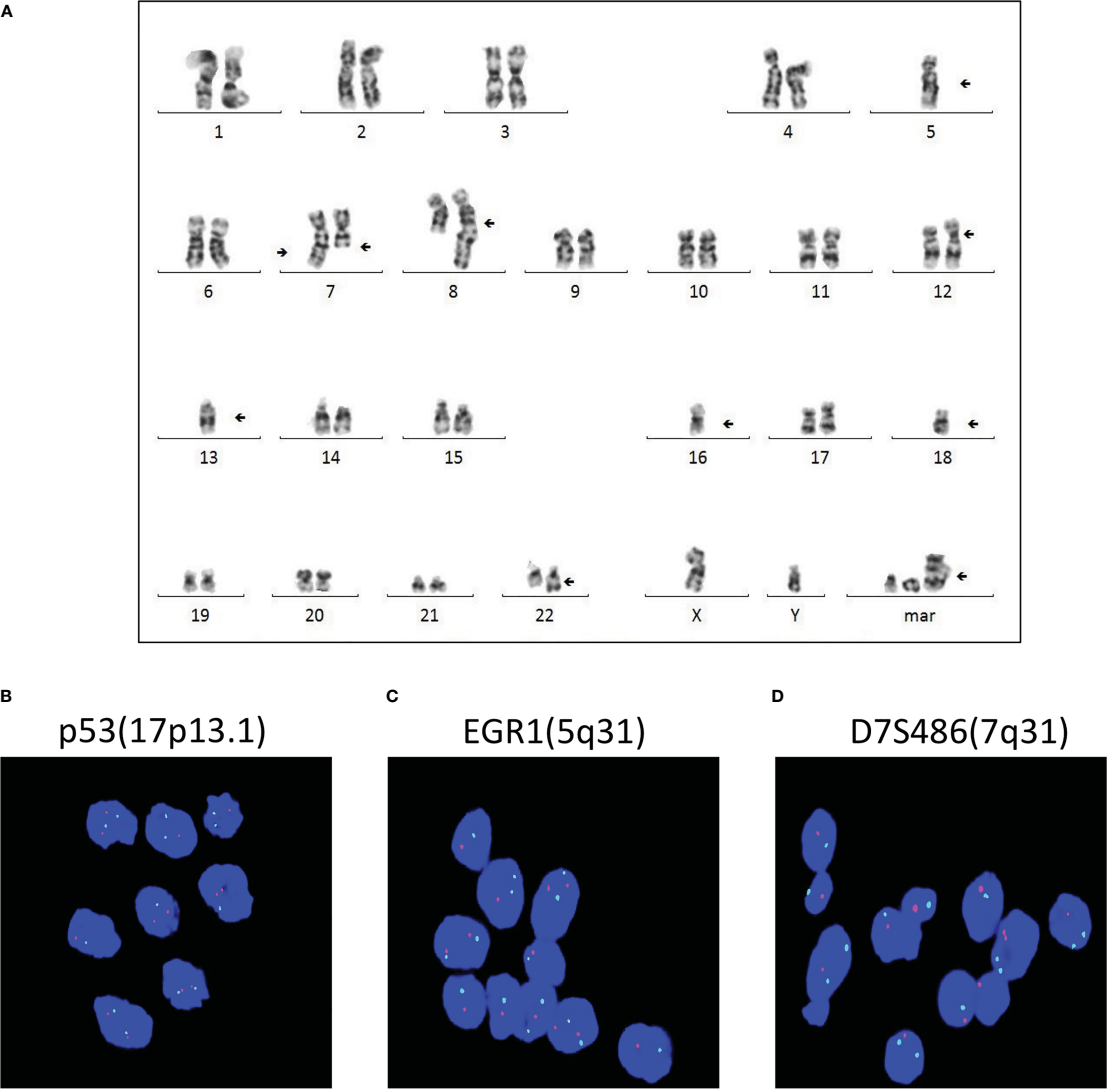
Multiple chromosomal abnormalities of bone marrow and intracranial GCT. **(A)** A representative karyotype from the diagnostic bone marrow specimen, which included 43-46, XY, +1, add (1) (p22), add (4)(q35), -5, -7, add (7)(q36), del (7)(q31), add (8)(q24), add (12)(p11), del (12)(q24), -13, -14, -16, -18, add (22)(q13), and +1-4mar[cp14]/46,xy[2]. **(B–D)** FISH analysis was carried out to detect +8, +12, -5, -7, and P53 gene of the intracranial germ cell tumors.

The cytogenetic study revealed multiple chromosomal abnormalities: 43-46, XY, +1, add (1)(p22), add (4)(q35), -5, -7, add (7)(q36), del (7)(q31), add (8)(q24), add (12)(p11), del (12)(q24), -13, -14, -16, -18, add (22)(q13), and +1-4mar[cp14]/46,xy[2] (**Figure 3A**). This complex karyotype was further confirmed by CytoScan 750K Cytogenetics Array analysis, with multiple abnormalities in chromosome regions (**Table 1**). Meanwhile, the molecular test for AML mutational study was negative. To investigate the relationship of these two diseases, we performed FISH analysis on the tissue of the intracranial GCT for +8, +12, -5, -7, and P53 gene. The results showed that the GCT cells had the deletion of p53(17q13.1), EGR1(5q31), and D7S486(7q31) (**Figures 3B–D**), which were also detected in the blast cells of the bone marrow.

**Table 1:** Cytogenetics array analysis of bone marrow cells.

chromosome	Abb ration	length	location
**1**	Whole chromosome duplication		
**5**	Long arm partial deletion	100.7Mb	5q14.1q35.3(79,967,067-180,715,096)
**8**	Long arm partial repeat	41.86Mb	8q22.2q24.3(99,945,588-141,805,646)
**9**	Long arm CN-LOH		9q21.11q34.3(71,013,799-140,895,240)
**12**	short arm partial deletion	22.87Mb	12p13.31p11.21(9,011,909-31,881,141)
**13**	Whole chromosome deletion		
**16**	short arm partial deletion	31.68Mb	16p13.3p11.2(85,880-31,761,166)
**17**	Whole chromosome CN-LOH		
**21**	Whole chromosome repeat		
**22**	Whole chromosome with complicated change (duplication to triplication, partial region with CN-LOH)		

(Copy neutral loss of heterozygosity-LOH); (loss of heterozygosity, OH).

### Acute bone marrow necrosis led to death

In May 2013, the patient was diagnosed and started on induction chemotherapy after counseling. The chemotherapy regimen comprised decitabine 20mg/m^2^ from day 1 to day 5, Granulocyte-Colony Stimulating Factor (G-CSF) 250μg from day 1 to day 5, cytarabine 10mg/m^2^ q12h from day 1 to day 5, and aclarubicin 20mg at day1, day 3, and day 5. A bone marrow aspiration and flow cytometry analysis for minimal residual disease were performed to evaluate the efficacy two days after the cessation of chemotherapy, which revealed no blast cells within 3×10^5^ cells. In addition, he was infused with peripheral blood lymphocyte from his father 48 hours after completion of the chemotherapy, with a total dose of CD3+ lymphocyte of 1.56×10^8^/kg. He achieved complete remission one month later, with normalization of the full blood count and bone marrow.

Afterward, he underwent consolidation therapy consisting of one cycle of DAE (daunorubicin, cytarabine, and etoposide) regimen and two cycles of MA (mitoxantrone and cytarabine) regimen. We strongly recommended allogeneic hematopoietic stem cell transplantation (HSCT) as a follow-up treatment for the patient, considering the high risk of leukemia relapse. However, despite explaining the risks to his family members, they were not willing to pursue HSCT.

**Figure 4:**
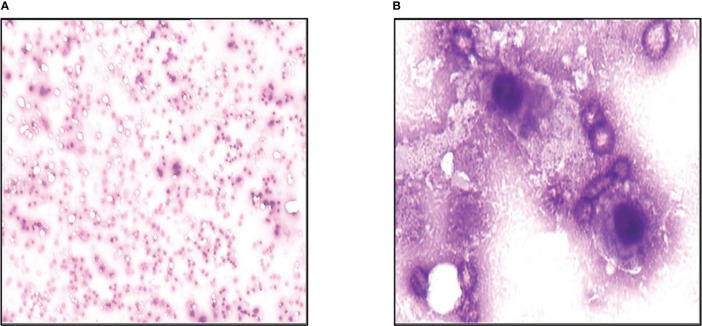
Acute bone marrow necrosis led to death. **(A)** Peripheral blood smear examination of the case by microscope. **(B) **Bone marrow smear examination of the patient using microscope.

On November 12^th^, 2013, he developed a fever. His full blood count showed severe pancytopenia, with white blood cells count of 2.5×10^9^/L, hemoglobin 51g/L, and platelet count of 6×10^9^/L. He was promptly admitted and started on broad-spectrum antibiotics. A peripheral blood film and bone marrow examination were performed, which showed numerous necrotic cells on both the peripheral blood (**Figure 4A**) and bone marrow smears (**Figure 4B**). This appearance was compatible with acute bone marrow necrosis. Unfortunately, the patient succumbed to the illness on 28th November 2013 despite receiving intensive antibacterial therapy and supportive care.

### Mutation landscape of intracranial GCT and AML-M7 samples of this patient

**Figure 5:**
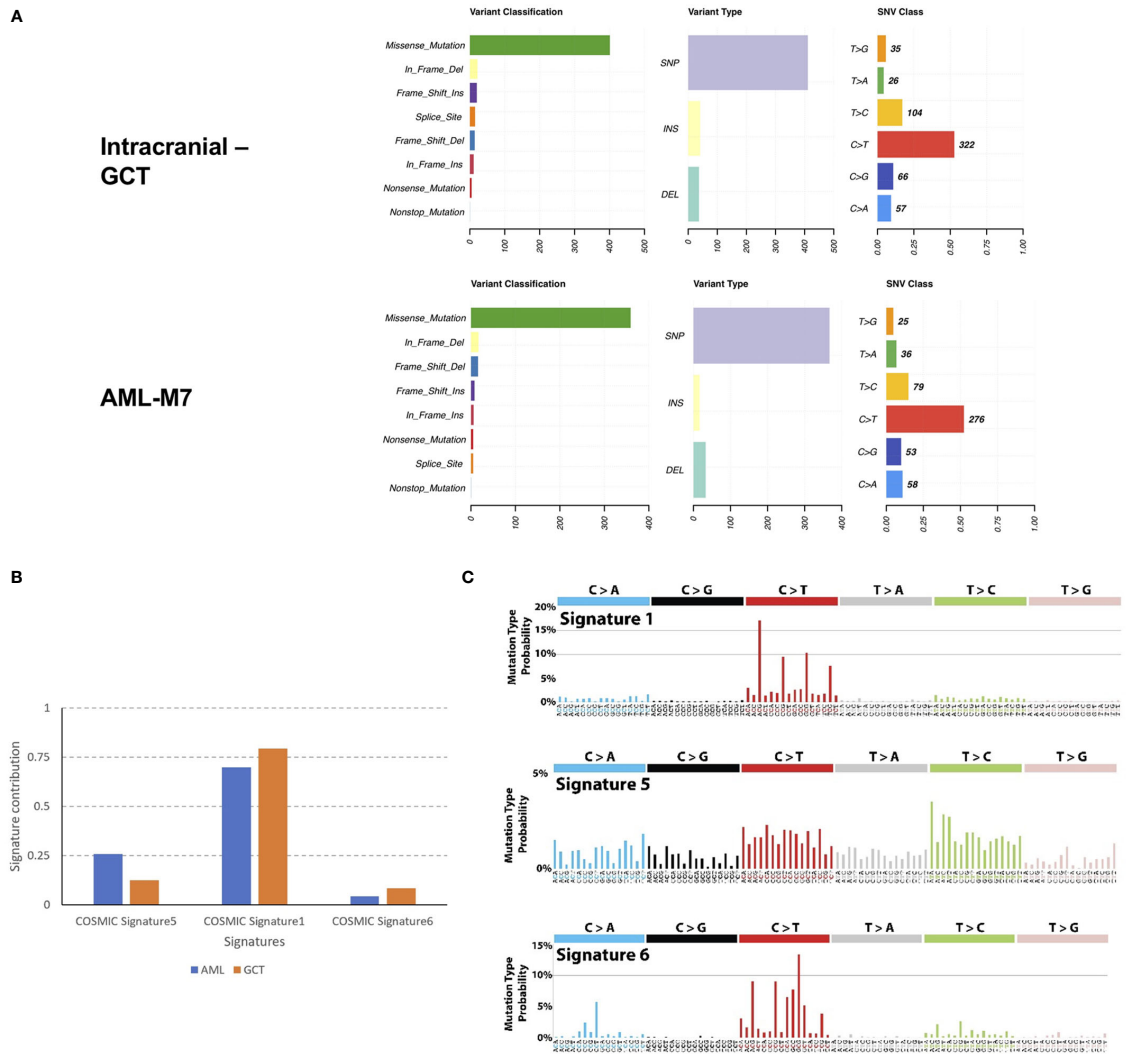
Mutation landscape of intracranial GCT and AML-M7 samples of this patient. **(A)** Formalin-fixed paraffin-embedding of intracranial GCT sample and AML-M7 bone marrow sample of this case were used for the WES on Illumina platform and variant analysis for classification, type and SNV class were performed. **(B, C)** Mutation signatures analysis compared with COSMIC signatures for GCT and AML-M7 bone marrow samples.

In order to investigate the relationship between intracranial GCT and AML-M7 in more detail, formalin-fixed paraffin-embedding (FFPE) samples from both types of cancer were used for WES on the Illumina platform. The sequencing data obtained was 9 Gb and 39 Gb for intracranial GCT and AML-M7 samples respectively, with a coverage of 56X and 191X. The coverage of sequencing data was shown in **Table S1**, and fraction of target bases analysis showed the sequencing worked well for next study (**Figure S2**). Due to the absence of normal tissue of germ cell tumor biopsy and bone marrow biopsy in complete remission for next-generation sequencing testing, a mutation was considered somatic if the frequency of the variant was less than 0.5% at several databases, which could exclude a vast majority of germline variants, but not all. After filtering out potential germline variants, 688 (647 SNVs and 41 indels) and 577 (560 SNVs and 17 indels) mutations were identified in intracranial GCT and AML-M7 samples respectively. For variant classification analysis, missense mutations were found to be the main type of mutation for both types of cancer, followed by in-frame deletion, frame-shift insert, splice site, frame-shift deletion, in- frame insert, nonsense mutation, and nonstop mutation. This indicated that the mutational landscape was similar between intracranial GCT and AML-M7. For variant type analysis, SNPs were found to be the main type of mutation in both intracranial GCT and AML-M7. However, the trends for insert type and deletion type were not consistent. The most common SNV type was C>T, accounting for 53% and 52% of mutations in intracranial GCT and AML-M7 respectively (**Figure 5A**). We compared the mutation signatures with COSMIC signatures using maftools package (19); three COSMIC signatures were identified in these two samples (AML and GCT, **Figure 5B**). According to the contribution of each signature, the C>T mutational signature was mainly corresponding to COSMIC Signature1 (**Figures 5B, C**). COSMIC Signature1 has been found in most cancer samples; it is the result of an endogenous mutational process initiated by spontaneous deamination of 5-methylcytosine (20, 21). Notably, COSMIC Signature1 was identified as the main signature in patients with AML in previous studies (20), which was consistent with the mutation signatures in this study.

### AML/Pan cancer driver genes were shared by intracranial GCT and AML-M7 samples of this patient

**Figure 6:**
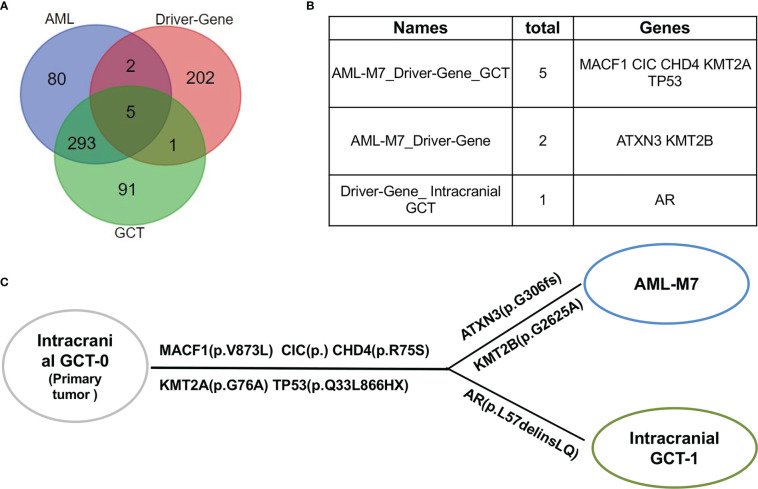
AML/Pan cancer driver genes were shared by intracranial GCT and AML-M7 samples of this patient. **(A)** The Venn diagram of the numbers of mutation genes shared by AML-M7, intracranial GCT, and tumor driver genes. **(B)** The specific genes shared by AML-M7, intracranial GCT, and tumor driver genes related to **(A)**. **(C)** Diagram showing the evolutionary relationship between intracranial GCT and AML-M7.

Upon further analysis, it was found that there were 380 mutant genes in intracranial GCT and 390 mutant genes in AML-M7 samples. Interestingly, 298 genes were found to be shared by both samples (**Figure 6A**). In order to determine the evolutionary relationship between the two samples, the driver gene dataset (22) for each sample was annotated. Out of 380 mutant genes in AML-M7 sample, six were identified as cancer driver genes, while seven were identified as cancer driver genes out of 390 mutant genes in intracranial GCT sample (**Figure 6A**). Five driver genes (MACF1, CIC, CHD4, KMT2A, and TP53) were mutated in both samples at the same time (**Figure 6B**), and the mutation sites for these five genes were identical (**Tables 2**). Among them, TP53 is a well-known driving gene for leukemia, while the other four genes are pan-cancer driver genes (23, 24). Based on the occurrence and the mutation site of the driving genes, we constructed the evolutionary relationship between the two samples. From the evolutionary map, it was observed that the two tumors belong to the same origin and began to differentiate in the later stage (**Figure 6C**).

**Table 2:** Mutation sites of intracranial GCT sample and AML-M7 sample.

Chromo some	Mutation site	Gen e	Mutation type	Mutation Ref-Alt	AA change	GCT Tumor type	Allele frequency GCT	Allele frequency AML-M7
chr1	3976600 2	MAC F1	missense SNV	G-C	V873L	GCT & AML- M7	0.27	0.70
chr11	1183074 54	KMT 2A	missense SNV	G-C	G76A	GCT & AML- M7	0.4	0.60
chr12	6711341	CHD 4	missense SNV	G-T	R75S	GCT & AML- M7	0,55	0.56
chr14	9253735 4	ATX N3	frame shift insertion	TGCTGCT GCTGCTG CTGCTGC TGCTGCT GCTGCTG CTGCTG	G23fs	AML-M7	ND	0.42
chr17	7578437	TP53	Stop gain	G-A	Q33X	GCT & AML- M7	0.91	0.98
chr19	4279551 7	CIC	missense SNV	T-A	L866H/L1775H	GCT	0.45	0.68
chr19	3622918 4	KMT 2B	missense SNV	G-C	G2625A	AML-M7	ND	0.36
chrX	6676515 8	AR	non-frame shift insertion	GCAGCA GCAGCA GCAGCA GCAGCA GCA	L57delin sLQQQ QQQQ QQ	GCT	1.00	ND

## Discussion

Nichols et al. reported on two men who had primary mediastinal germ-cell tumor and later developed acute megakaryocytic leukemia (7). Since then, several similar cases have been reported (25, 26). In most cases, patients with mediastinal GCT develop the hematological malignancy several months later, with a median interval of 6 months between the two diagnoses (15). However, in a small proportion of patients, both malignancies are diagnosed concurrently. Herein, we present a rare case of acute megakaryocytic leukemia that occurred shortly after the diagnosis and treatment of an intracranial GCT, which has never been reported before. Our patient was diagnosed with acute megakaryocytic leukemia 7 months after the first chemotherapy for his intracranial GCT, which suggested less possibility for the leukemia as a therapy-related AML.

The prognosis for patients with AML-M7 following GCT is extremely poor, with a majority of patients being refractory to chemotherapy and some even succumbing to the disease before the treatment can be initiated (27). However, accumulating evidence has suggested that decitabine, a demethylating agent, can improve the chemosensitivity and immunogenicity of malignant cells by inducing the expression of certain genes (28–30). In this patient, decitabine-based chemotherapy followed by haploidentical lymphocyte infusion was applied and achieved complete remission. The only curative therapy for AML-M7 after intracranial GCT is allogeneic hematopoietic stem cell transplantation after complete remission, as the risk of relapse is extremely high. However, our patient and his family members declined this option after counseling and explanation. Unfortunately, the patient presented with spiking fever and pancytopenia one month after completion of the treatment. It was impossible to ascertain the disease status because of the severe necrosis of the bone marrow.

There are reports of common mutational abnormalities in the cancerous cells of GCT and AML-M7. Specifically, the most common abnormal gene identified was isochromosome 12 (31, 32). Through cytogenetic study and CytoScan 750K Cytogenetics Array analysis, multiple chromosomal abnormalities were detected. Based on these shared chromosomal abnormalities, we proposed that both the AML-M7 and intracranial GCT in this patient originated from the same progenitor cell. In order to further prove this hypothesis, WES and gene mutation analysis were carried out, revealing that 298 mutation genes were shared by intracranial GCT and AML-M7 samples in our patient. Amongst these, MACF1 (33), CIC (34), CHD4 (35), KMT2A (36), and TP53 genes were found to be mutated, which are all known to be cancer driver genes. Based on the occurrence and mutation sites, an evolutionary relationship was constructed between the two samples, revealing that the two tumors likely shared the same origin and began to differentiate in the later stage of their development. Overall, our findings provide support for the theory that aAML-M7 and intracranial GCT may originate from the same progenitor cells.

## Data availability statement

The original contributions presented in the study are included in the article/**Supplementary Material**. Further inquiries can be directed to the corresponding author.

## Ethics statement

The studies involving human participants were reviewed and approved by Ethics Committee of Naval General Hospital. Written informed consent was obtained from the minor(s)’ legal guardian/next of kin for the publication of any potentially identifiable images or data included in this article.

## Author contributions

L-XW designed and performed the project, wrote and revised the manuscript, and supervised the project. W-JL analyzed the data and wrote and revised the manuscript. CC performed the gene mutation analysis. Y-HJ, W-SL, FY, and H-YN performed the project and analyzed the data. All authors contributed to the article and approved the submitted version.
