# Supplementary material for: Gene mutation landscape of a rare patient with acute megakaryoblastic leukemia after treatment of intracranial germ cell tumor
Source: Front Oncol. 2023 May 9;13:1093434. doi: 10.3389/fonc.2023.1093434 (PMC10203525; doi:10.3389/fonc.2023.1093434)
Supplement: Supplementary file 1 [file Presentation_1.pptx]

## Slide 1
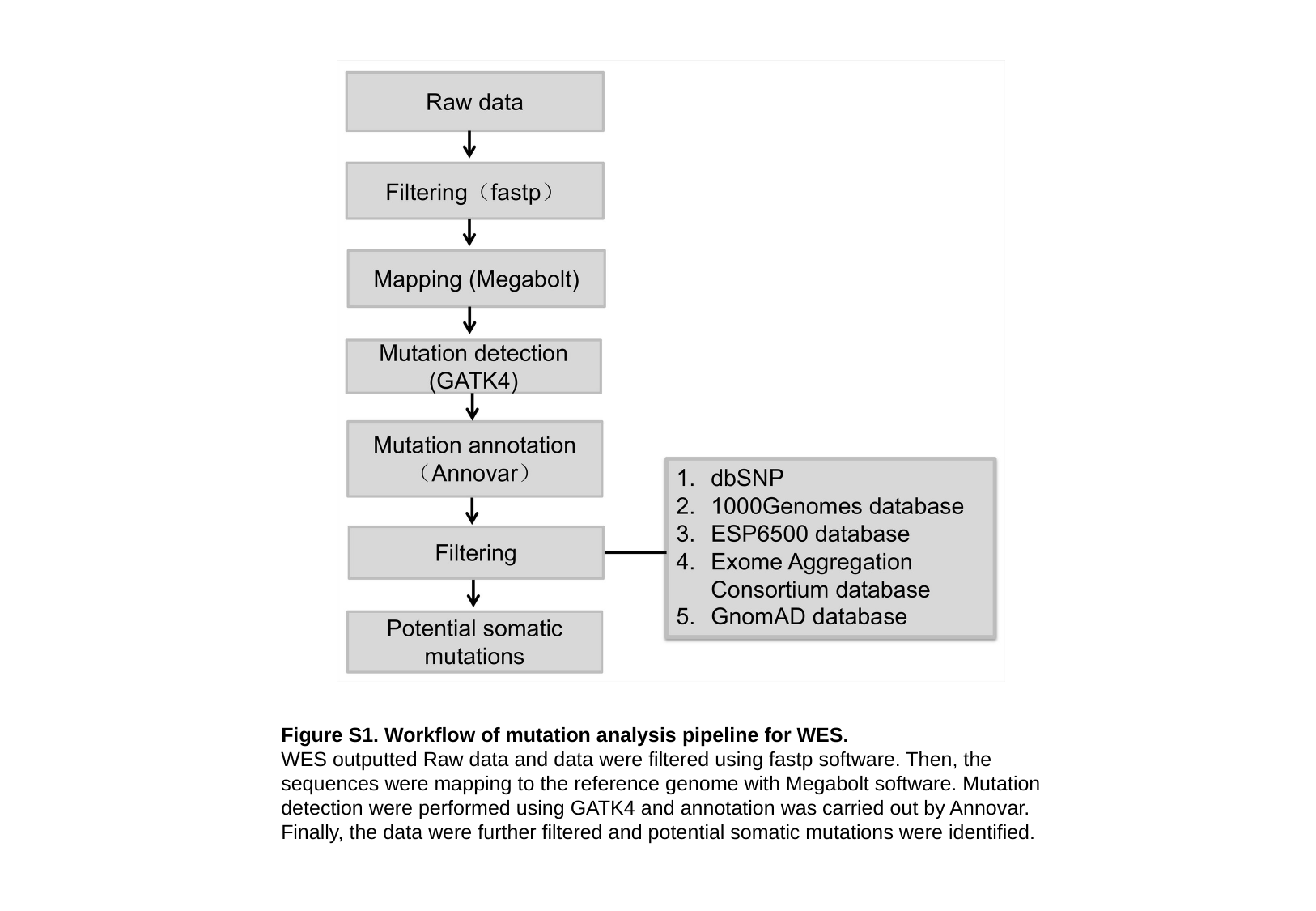

Figure S1. Workflow of mutation analysis pipeline for WES.
WES outputted Raw data and data were filtered using fastp software. Then, the sequences were mapping to the reference genome with Megabolt software. Mutation detection were performed using GATK4 and annotation was carried out by Annovar. Finally, the data were further filtered and potential somatic mutations were identified.

## Slide 2
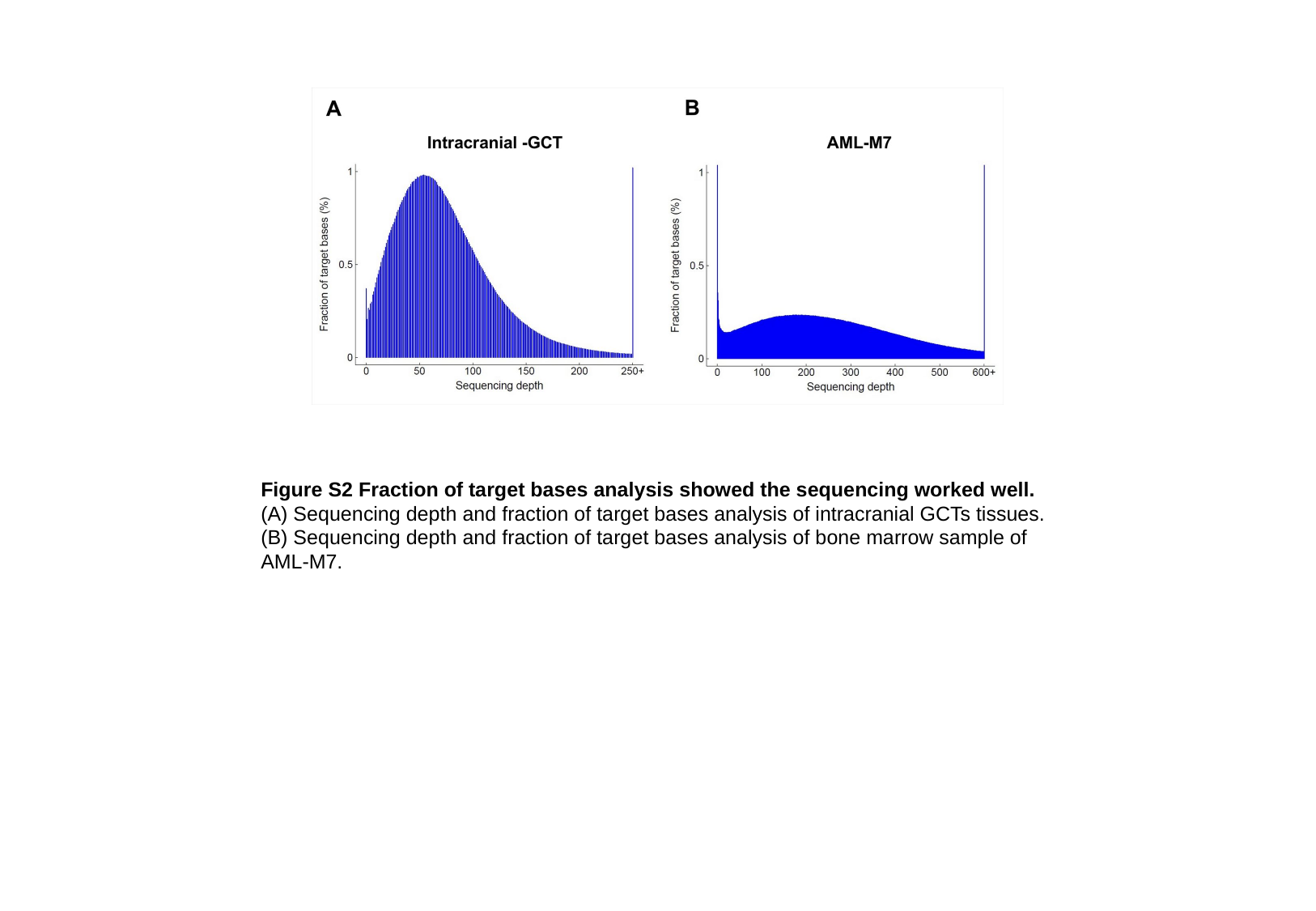

Figure S2 Fraction of target bases analysis showed the sequencing worked well.
(A) Sequencing depth and fraction of target bases analysis of intracranial GCTs tissues. (B) Sequencing depth and fraction of target bases analysis of bone marrow sample of AML-M7.

## Slide 3
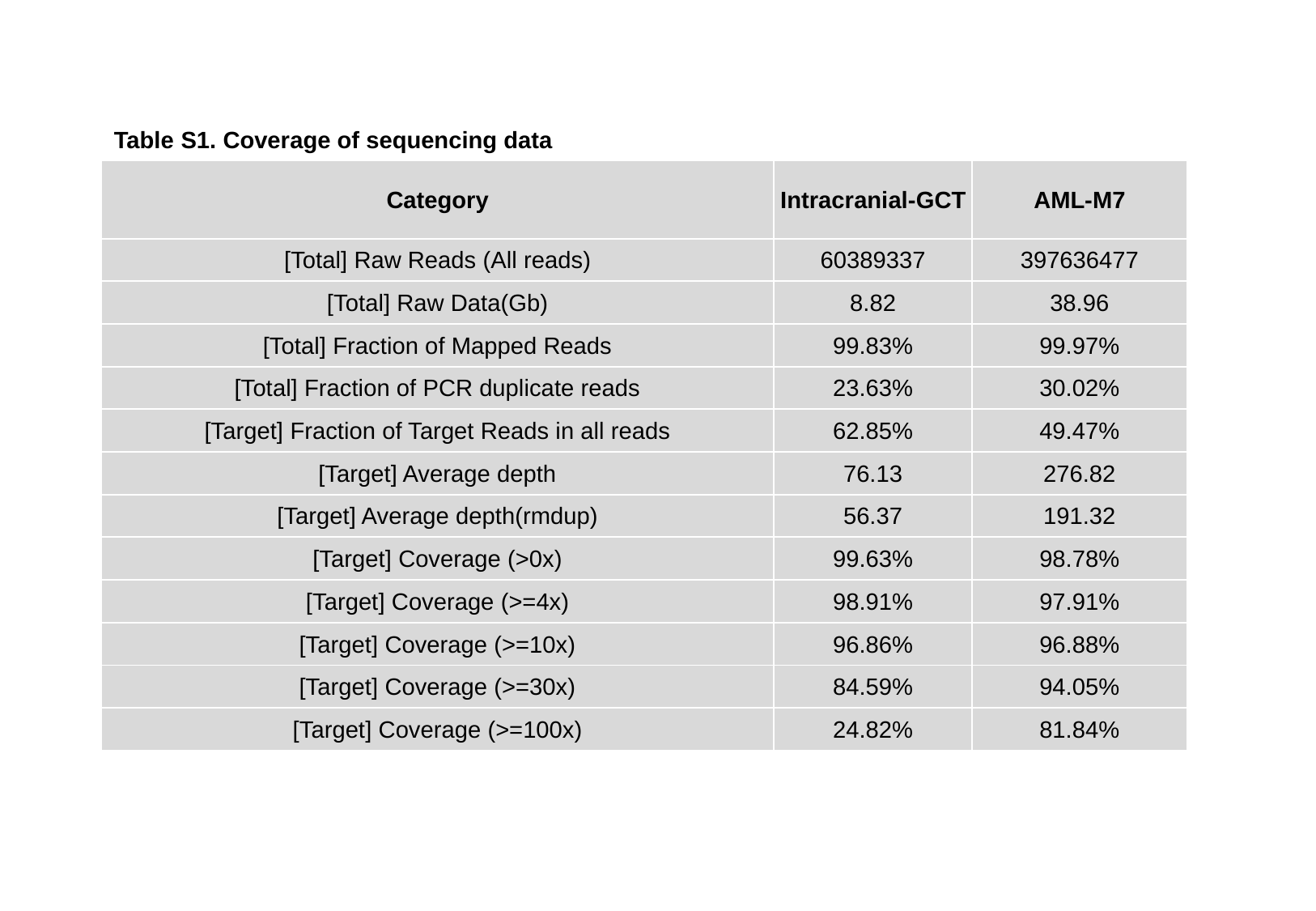

Table S1. Coverage of sequencing data
| Category | Intracranial-GCT | AML-M7 |
| --- | --- | --- |
| [Total] Raw Reads (All reads) | 60389337 | 397636477 |
| [Total] Raw Data(Gb) | 8.82 | 38.96 |
| [Total] Fraction of Mapped Reads | 99.83% | 99.97% |
| [Total] Fraction of PCR duplicate reads | 23.63% | 30.02% |
| [Target] Fraction of Target Reads in all reads | 62.85% | 49.47% |
| [Target] Average depth | 76.13 | 276.82 |
| [Target] Average depth(rmdup) | 56.37 | 191.32 |
| [Target] Coverage (>0x) | 99.63% | 98.78% |
| [Target] Coverage (>=4x) | 98.91% | 97.91% |
| [Target] Coverage (>=10x) | 96.86% | 96.88% |
| [Target] Coverage (>=30x) | 84.59% | 94.05% |
| [Target] Coverage (>=100x) | 24.82% | 81.84% |
